# Preparation and Stability Study of Co-Encapsulated Particles of Curcumin or Quercetin with *Lactobacillus rhamnosus* GG

**DOI:** 10.3390/foods15111910

**Published:** 2026-05-28

**Authors:** Xiangyu Yang, Jinxiu Zhang, Zizhen Ren, Xinzhong Hu, Zhen Ma

**Affiliations:** College of Food Engineering and Nutritional Science, Shaanxi Normal University, Xi’an 710062, China; joe_franklyn@foxmail.com (X.Y.); hxinzhong@snnu.edu.cn (X.H.)

**Keywords:** co-encapsulation, curcumin, quercetin, *Lactobacillus rhamnosus* GG, debranched starch

## Abstract

This study developed and characterized a hierarchical co-encapsulation system for the delivery of hydrophobic polyphenols (curcumin or quercetin) and the probiotic *Lactobacillus rhamnosus* GG (LGG). The system was constructed through the self-assembly of zein to form a hydrophobic core for the polyphenols, followed by complex coacervation with debranched starch and chitosan to form an outer hydrophilic layer for LGG. Structural analyses using X-ray diffraction, Fourier transform infrared spectroscopy, scanning electron microscopy, and confocal laser scanning microscopy verified the successful formation of the core–shell structure and the transformation of crystalline polyphenols into an amorphous state. The co-encapsulated particles exhibited high encapsulation efficiency (>95% for both polyphenols and LGG) and encapsulation yield (>80% and >75%, respectively). The encapsulation significantly enhanced the antioxidant capacity of both polyphenols in DPPH and ABTS assays, with co-encapsulation of LGG providing a further enhancement. Moreover, the hierarchical structure effectively protected LGG, markedly improving its survival under simulated gastrointestinal conditions and during storage at 4 °C and −20 °C, with quercetin offering stronger protection than curcumin. These findings demonstrate that this co-encapsulation strategy delivers simultaneous protection to both probiotics and polyphenols, providing a robust approach for improving the stability and functionality of multiple bioactive components.

## 1. Introduction

Functional bioactive components play a critical role in maintaining human health. However, the efficacy of many of these components is often compromised by their poor stability during processing and under gastrointestinal conditions. Factors such as light, heat, oxygen, and pH can induce degradation, reduce bioavailability, and lead to the formation of undesirable metabolites. To overcome these limitations, growing interest has been directed toward encapsulation strategies designed to protect these compounds and enable their targeted delivery. A notable example is the probiotic *Lactobacillus rhamnosus* GG (LGG), recognized for its ability to modulate intestinal flora, strengthen immune responses, and improve lipid metabolism [1]. Despite these benefits, LGG is highly sensitive to environmental stresses such as gastric acid, bile salts, temperature, and oxygen, which significantly reduce its survival through the gastrointestinal tract, thereby limiting its probiotic functions.

Starch, a biodegradable and biocompatible polymer, is widely used in encapsulation systems due to its low cost, simple preparation, and good thermal stability. In particular, debranched starch, produced by enzymatic treatment of amylopectin with pullulanase to hydrolyze α-1,6-glycosidic bonds, exhibits enhanced enzyme resistance and superior thermal properties compared to native starch [2]. Through retrogradation, debranched starch can reassemble into porous or spherical particles, making it suitable for constructing self-assembled micelles or nanocapsules for the targeted delivery of functional ingredients [3]. The structural characteristics of debranched starch, such as molecular weight distribution, chain length, and crystallinity, are influenced by the degree of debranching and retrogradation conditions, which in turn govern its performance as an encapsulation matrix.

Lentil starch possesses a high amylose content and a favorable chain-length distribution after debranching, which promotes the formation of stable helical structures and enhances its ability to form retrograded particles suitable for probiotic encapsulation. Compared to systems encapsulating a single ingredient, co-encapsulating multiple bioactive substances within one system offers the potential for enhanced interactions among functional components, thereby enhancing health benefits and meeting diverse and personalized nutritional needs. A particularly promising strategy is the integration of polyphenols and probiotics within a single encapsulation system, which not only provides mutual protection but may also enhance their respective biological activities [4,5]. For example, polyphenols such as curcumin and quercetin exhibit strong antioxidant activity and can act as prebiotics to promote the growth of beneficial bacteria. Studies shown that co-encapsulation of probiotics with polyphenols improves both the survival of the probiotics under gastrointestinal conditions and the chemical stability of the polyphenols [6,7,8].

Complex co-assembly is an effective microencapsulation technique for entrapping functional ingredients, offering enhanced stability and controlled release. Zein, a hydrophobic plant protein, is widely recognized for its biocompatibility, safety, and solubility in 60–95% ethanol solutions. Through a solvent displacement process, zein can form hydrophobic cavities suitable for loading lipophilic bioactive compounds [9,10]. However, particles fabricated solely from zein often exhibit limited colloidal and structural stability. To improve their stability, zein is frequently integrated with complementary biopolymers, such as debranched starch or chitosan, to enhance mechanical strength and functional performance [11,12]. The complexation of zein with water-soluble polysaccharides via phase separation enables the encapsulation of hydrophilic bioactive substances. This approach facilitates the construction of a hierarchical delivery vehicle capable of co-encapsulating two or more functional ingredients. Such a design not only improves the protection of both hydrophobic and hydrophilic bioactive compounds but may also improve their combined functional efficacy [4,5].

In this study, a co-encapsulation system containing either curcumin (Cur) or quercetin (Que) together with LGG was developed. The primary objectives were to evaluate the protective effects of the hierarchical particulate structure on probiotic viability and to investigate the influence of the encapsulated polyphenols on LGG. To address this challenge, the present study developed a hierarchical co-encapsulation system combining zein self-assembly with debranched starch and chitosan complex coacervation, and systematically evaluated its structural characteristics, encapsulation efficiency, antioxidant capacity, and protective effects on LGG under simulated gastrointestinal conditions and during storage. The effects of debranching degree on the encapsulation efficiency, gastrointestinal tolerance, and storage stability of LGG were systematically investigated. This study is expected to establish a novel methodological strategy for constructing advanced co-encapsulation systems with potential applications in functional foods and nutraceuticals.

## 2. Materials and Methods

### 2.1. Materials

Lentils were provided by Qichen Agricultural Products Co., Ltd. (Heze, Shandong, China). *Lactobacillus rhamnosus* GG (LGG, strain ATCC 53103) was obtained from the American Type Culture Collection (Manassas, VA, USA). Pullulanase (E.C. 3.2.1.41, activity ~1498 NPUN/g), curcumin, and quercetin (HPLC grade) were supplied by Sigma-Aldrich (St. Louis, MO, USA). Zein (food grade) was purchased from Shanghai Macklin Biochemical Technology Co., Ltd. (Shanghai, China), chitosan (food grade, deacetylation degree ≥90%) was obtained from Shanghai Lanji Technology Development Co., Ltd. (Shanghai, China). All other chemicals and solvents used were of analytical grade.

### 2.2. Preparation of Bacterial Suspension and Growth Assay

LGG from a −80 °C stock was thawed, inoculated into MRS broth, and activated through two generations of anaerobic culture at 37 °C for 24 h using an anaerobic incubator (YQX-II, Shanghai CIMO Medical Instrument Co., Ltd., Shanghai, China) with an atmosphere of 85% N_2_, 10% H_2_, and 5% CO_2_. The activated LGG was then inoculated (2%, *v*/*v*) into fresh MRS broth and cultured anaerobically at 37 °C for 12 h to the late logarithmic phase. Cells were harvested by centrifugation at 4400 *g* for 10 min at 4 °C using a high-speed refrigerated centrifuge (TGL-20M, Changsha Xiangyi Centrifuge Co., Ltd., Changsha, China), washed three times with sterile saline solution (0.9%, *w*/*v*), and resuspended to a final concentration of 10^8^ CFU/mL. The viable counts were confirmed by spread plating on MRS agar followed by anaerobic incubation at 37 °C for 48 h.

To evaluate the effect of polyphenols on LGG growth, activated LGG was inoculated into sterile MRS broth supplemented with curcumin or quercetin at concentrations of 0, 0.02, 0.05, 0.1, 0.2, and 0.5% (*w*/*v*). The polyphenols were first dissolved in a minimal volume of dimethyl sulfoxide (DMSO), with a final DMSO concentration below 0.5% (*v*/*v*) in the broth, to ensure solubilization before addition to the culture medium. The cultures were incubated anaerobically at 37 °C for 24 h, after which viable cell counts were determined as described above.

### 2.3. Preparation of Debranched Starch (DBS)

Lentil starch was isolated as previously described [13]. Briefly, lentils were soaked overnight, homogenized, and passed through a 200-mesh sieve. The slurry was centrifuged, and the starch layer was collected, repeatedly rinsed, and freeze-dried using a freeze dryer (LGJ-10C, Beijing Sihuan Scientific Instrument Co., Ltd., Beijing, China). The native starch was gelatinized in boiling water at 20% (*w*/*v*) concentration and autoclaved at 121 °C for 30 min. An equal volume of acetate buffer (200 mmol/L, pH 5.5) was added, and the dispersion was homogenized using a high-speed disperser (XHF-D, Ningbo Xinzhi Biotechnology Co., Ltd., Ningbo, China). The particle size of the resulting nanoparticles was verified by dynamic light scattering (DLS) using a Zetasizer Nano ZS (Malvern Panalytical, Malvern, UK), which showed a mean hydrodynamic diameter of 150–200 nm. After cooling to 55 °C, pullulanase was added at 30, 60, and 90 NPUN/g starch. The reaction proceeded at 55 °C with stirring for 8 h, followed by enzyme inactivation in a boiling water bath for 15 min. The supernatant containing the debranched starch (DBS) was collected by centrifugation. A portion was used directly for encapsulation, while the remainder was stored at 4 °C for 24 h, freeze-dried, and sieved (100-mesh) to obtain DBS powders, designated as DBS30, DBS60, and DBS90. Untreated lentil starch (UDBS) served as a control.

### 2.4. Preparation of Encapsulated Particles

#### 2.4.1. Zein-Polyphenol Complexes (ZC/ZQ)

This study developed a hierarchical co-encapsulation system through the self-assembly of zein to form a hydrophobic core for the polyphenols (curcumin or quercetin), followed by complex coacervation with debranched starch and chitosan to form an outer hydrophilic layer for the probiotic *Lactobacillus rhamnosus* GG (LGG). The detailed preparation procedures are described below. Zein (2.0 g) was dissolved in 100 mL of 80% (*v*/*v*) ethanol under magnetic stirring for 1 h. Curcumin or quercetin was added to a final concentration of 0.05% (*w*/*v*), and stirring continued for another hour to obtain Zein-Cur (ZC) and Zein-Que (ZQ) stock solutions, which were stored at 4 °C in the dark. Following established methods with modifications [14,15], the stock solutions were added dropwise to deionized water (1:4, *v*/*v*) under magnetic stirring. Ethanol was removed by rotary evaporation at 40 °C for 30 min, and the volume was adjusted with water to form Zein-polyphenol complex nanoparticle dispersions. For dry ZC/ZQ complex powders, the dispersions were freeze-dried.

#### 2.4.2. Co-Encapsulated Particles (ZCLS/ZQLS)

The Zein-polyphenol nanoparticle dispersion (10 mL) was mixed with 10 mL of LGG suspension (10^8^ CFU/mL). The DBS supernatant (80 mL) was added, followed by chitosan (dissolved in 1% *v*/*v* acetic acid solution), to a final concentration of 0.1% (*w*/*v*). The mixture was stirred for 2 h and then retrograded at 4 °C for 24 h. The resulting co-encapsulated probiotic particles, designated as Zein-Cur-LGG-DBS (ZCLS) and Zein-Que-LGG-DBS (ZQLS), were obtained by freeze-drying and stored at −20 °C. These particles contained LGG, polyphenols, and were embedded within the DBS-chitosan matrix. Control particles, including Zein-Cur-DBS (ZCS, containing curcumin but no LGG), Zein-Que-DBS (ZQS, containing quercetin but no LGG), and Zein-LGG-DBS (ZLS, containing LGG but no polyphenol), were prepared similarly for comparative studies [16].

### 2.5. Encapsulation Efficiency (EE) and Yield (EY)

To determine the encapsulation efficiency (EE) and encapsulation yield (EY), the freeze-dried co-encapsulated or control particles (0.1 g) were first washed with saline. The supernatant was collected for plating to quantify surface-adherent bacteria (S). The washed particles were dissolved in phosphate buffer (0.1 M, pH 7.0) at 37 °C for 2 h to release the encapsulated bacteria. The viable count obtained from this step was designated as R, representing the number of viable bacteria encapsulated within the particles. Total viable bacteria (T) in the particles was calculated as the sum of the surface-adherent viable bacteria (S) and the viable bacteria encapsulated within the particles (R) EE and EY were then calculated as follows:

EE (%) = (R/T) × 100

EY (%) = (T/W) × 100

where R is the number of viable bacteria encapsulated within the particles, S is the number of surface-adherent viable bacteria, T is the sum of S and R, and W is the number of viable bacteria initially added during the encapsulation process.

The content of curcumin and quercetin was quantified using UV-Vis spectroscopy (Multiskan Go Plate Reader, Thermo Fisher, Waltham, MA, USA) at λmax 418 nm and 375 nm, respectively, based on pre-established standard curves. Encapsulated particles were dissolved in 80% (*v*/*v*) ethanol under vortexing and mild heating (40 °C) to ensure complete release of polyphenols before measurement.

### 2.6. Structural Characterization

#### 2.6.1. X-Ray Diffraction (XRD)

The crystalline structure of the freeze-dried encapsulated particles, including the single encapsulated particles (ZC, ZQ, ZCS, ZQS, ZLS) and the co-encapsulated particles (ZCLS, ZQLS), as well as the raw materials (Cur, Que, Zein, DBS) was analyzed using an X-ray diffractometer (D/Max2550VB+/PC, Rigaku, Tokyo, Japan) with Cu-Kα radiation. Samples were scanned from 4° to 40° (2θ) at a step size of 0.02° and a rate of 5°/min. Relative crystallinity was calculated using Jade 6.5 software (Materials Data, Inc., Livermore, CA, USA).

#### 2.6.2. Fourier Transform Infrared Spectroscopy (FTIR)

FTIR spectra were acquired on a FTIR spectrometer (Tensor 27, Bruker, Ettlingen, Germany). Samples were mixed with KBr (1:100), pressed into pellets, and scanned from 4000 to 400 cm^−1^ at a resolution of 4 cm^−1^. The spectra were baseline-corrected, and the ratios of absorbance bands at 995/1022 cm^−1^ and 1047/1022 cm^−1^ were calculated to assess short-range molecular order.

#### 2.6.3. Environmental Scanning Electron Microscopy (ESEM)

The morphology of the samples, including raw materials (Cur, Que, natural zein, anti-solvent treated zein, DBS), single encapsulated particles (ZC, ZQ, ZCS, ZQS, ZLS), and co-encapsulated particles (ZCLS, ZQLS), was observed using an environmental scanning electron microscope (Quanta 200, FEI Company, Hillsboro, OR, USA). Samples were sputter-coated with gold and imaged at magnifications of ×2000 and ×10,000.

#### 2.6.4. Confocal Laser Scanning Microscopy (CLSM)

The internal structure of the assembled particles (ZCS, ZQS, ZLS, ZCLS, ZQLS) and the control samples (DBS, ZC, ZQ) was visualized using a confocal laser scanning microscope (FV1200, Olympus, Tokyo, Japan). To visualize spatial distribution of different components, samples were stained with Fluorescein Isothiocyanate (FITC; 0.2% in acetone) for starch and Rhodamine B (0.025% in aqueous solution) for protein, then destained with phosphate-buffered saline. According to established methodology [16], images were captured at excitation/emission wavelengths of 488/543 nm for FITC and Rhodamine B, respectively.

### 2.7. Antioxidant Capacity

#### 2.7.1. DPPH Radical Scavenging Activity

Prior to the antioxidant assays, the actual polyphenol content in the encapsulated particles was determined spectrophotometrically by dissolving a known mass of particles in 80% (*v*/*v*) ethanol and measuring the absorbance at the characteristic wavelengths for curcumin (418 nm) and quercetin (375 nm), as described in Section 2.5. For each assay, the amount of encapsulated particles was then adjusted to deliver the same quantity of polyphenols as the corresponding free polyphenol controls.

Sample solution was mixed with an equal volume of 2,2-Diphenyl-1-picrylhydrazyl (DPPH) in ethanol (200 μmol/L). After 30 min of incubation in the dark, absorbance was measured at 517 nm. Free polyphenols and their ethanol solutions served as controls. The radical scavenging rate was calculated as follows:

Scavenging rate (%) = [1 − (A_sample_/A_control_)] × 100

where A_sample_ is the absorbance of the sample with DPPH solution, and A_control_ is the absorbance of the DPPH solution without sample.

#### 2.7.2. ABTS Radical Scavenging Activity

ABTS (2,2′-azino-bis (3-ethylbenzothiazoline-6-sulfonic acid) radical working solution was prepared by reacting ABTS (14.4 mmol/L) with potassium persulfate (5.2 mmol/L) for 16 h, then diluted to an absorbance of 0.70 ± 0.02 at 734 nm. Sample solution (1 mL) was mixed with 4.5 mL of ABTS working solution, incubated for 6 min in the dark, and absorbance was measured at 734 nm. The scavenging rate was calculated using a formula similar to that for DPPH.

### 2.8. Simulated Gastrointestinal Tolerance

Simulated gastric juice (SGJ, pH 2.0) contained pepsin (3 g/L) in saline. Simulated intestinal juice (SIJ, pH 7.4) contained pancreatin (3 g/L) and bile salts (10 g/L) in phosphate buffer. Particles (0.1 g) were incubated in SGJ at 37 °C with shaking (120 rpm). At predetermined intervals (0, 0.5, 1, and 2 h), aliquots of the SGJ suspension (including both particles and any bacteria released into the medium) were withdrawn for viable counting to monitor LGG survival during the gastric phase. After 2 h of gastric digestion, the particles were collected by centrifugation (4400 *g* for 10 min), the supernatant was removed, and the particle precipitate was transferred to SIJ and incubated for up to 4 h. Similarly, aliquots were taken from the SIJ suspension at 1, 2, and 4 h for viable counting. Free LGG was used as a control.

### 2.9. Storage Stability

The storage stability of the encapsulated LGG was evaluated over a period of 5 weeks. The co-encapsulated particles (ZCLS and ZQLS) and control particles without polyphenols (ZLS) were stored at 4 °C and −20 °C to simulate refrigeration and frozen storage, respectively. The viability of LGG in the particles was assessed weekly by viable counts as described in Section 2.3. The survival rate was calculated using the following equation:

Survival rate (log CFU/g) = log_10_(*N*_0_) − log_10_(*N*ₜ)

where *N*_0_ is the initial viable count (CFU/g) at week 0 and *N*ₜ is the viable count at a given storage time t (weeks). This metric directly quantifies the loss of viability during storage and was used to evaluate the protective effect of the encapsulation matrices and the potential beneficial influence of the co-encapsulated polyphenols on the long-term stability of the probiotic.

### 2.10. Statistical Analysis

All experiments were performed in triplicate. Data are presented as mean ± standard deviation. Statistical significance (*p* < 0.05) was assessed using one-way ANOVA followed by DPS 7.05 software (Zhejiang University, Hangzhou, China).

## 3. Results and Discussion

### 3.1. Encapsulation Efficiency and Yield

Encapsulation efficiency (EE) and encapsulation yield (EY) are key indicators for assessing the performance of encapsulation systems. A high EE reflects successful incorporation of the core material into the wall matrix, whereas a high EY denotes effective retention of the initial core material throughout the encapsulation process [17]. Zein, a hydrophobic protein, is insoluble in water and anhydrous ethanol but readily soluble in 60–95% aqueous ethanol. This property allows it to undergo solvent displacement, a process that drives the self-assembly of zein into nanoparticles suitable for encapsulating non-polar compounds [9,10].

As presented in Table 1, zein-alone particles (ZC and ZQ) showed EE and EY values of 84.28% and 71.26% for curcumin, and 72.49% and 65.12% for quercetin, respectively. The notably higher encapsulation performance for curcumin may be attributed to its lower polarity and fewer hydroxyl groups, promoting stronger hydrophobic interactions with zein and thereby enabling more effective integration into the hydrophobic core. In contrast, the higher polarity of quercetin leads to a greater tendency for surface adsorption on zein nanoparticles, resulting in reduced EE and EY. These findings align with FT-IR and ESEM analyses, which revealed residual quercetin signals and surface adsorption in ZQ particles.

The encapsulation performance was markedly enhanced by further coating with debranched starch (DBS) and chitosan. The EE and EY for ZCS and ZQS increased to 96.21% and 80.27%, and 95.19% and 80.45%, respectively, demonstrating the successful development of a composite wall system that provides effective core protection. This is consistent with the results of Liang et al. [18], who confirmed that zein-chitosan nanoparticles constitute an efficient delivery platform. Furthermore, the co-encapsulated particles (ZCLS and ZQLS) achieved high EE and EY values for both LGG (94.87–95.09% and 78.39–79.72%) and polyphenols (96.56% and 81.73% for Cur; 95.64% and 80.79% for Que). These findings validate the successful establishment of a hierarchical encapsulation system capable of co-encapsulating both hydrophobic polyphenols and hydrophilic probiotics within a unified structure. These results confirm that the hierarchical co-encapsulation system effectively incorporates and retains both hydrophobic polyphenols and hydrophilic probiotics within a unified structure. The structural basis for this high encapsulation performance is further elucidated in the following sections.

### 3.2. Effect of Curcumin and Quercetin on LGG

**Figure 1:**
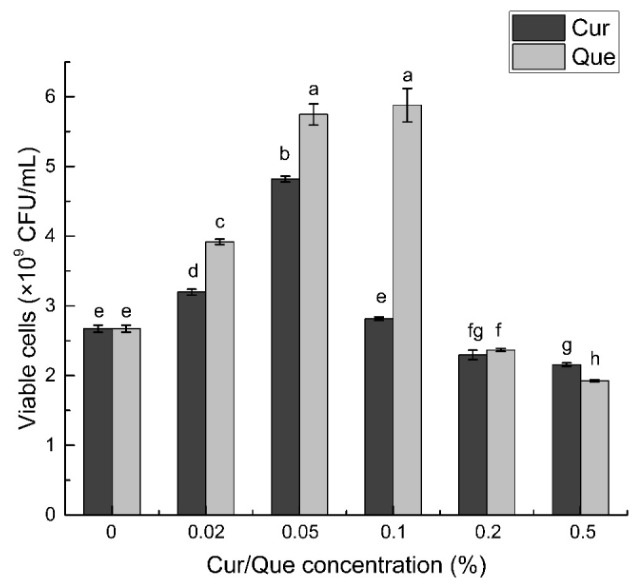
Effects of curcumin and quercetin on LGG growth. Different lowercase letters indicate significant differences (*p* < 0.05) as determined by one-way ANOVA followed by Duncan’s multiple range test.

Figure 1 shows the viable counts of LGG following supplementation with different concentrations of curcumin (Cur) or quercetin (Que), with each compound tested independently. The results indicate that at lower concentrations (0–0.1%), both polyphenols enhanced the growth of LGG, with quercetin demonstrating a stronger stimulatory effect than curcumin. This difference is likely due to the higher number of hydroxyl groups in quercetin, which may enhance its antioxidant capacity and prebiotic-like activity. These structural features may help alleviate oxidative stress and create a more favorable growth environment for the bacteria. In contrast, at higher concentrations (0.2% and 0.5%), both polyphenols inhibited LGG growth, possibly due to their inherent antimicrobial properties at elevated levels. The optimal growth-promoting effects were observed at 0.05% for curcumin and between 0.05% and 0.1% for quercetin. Therefore, a concentration of 0.05% was chosen for both polyphenols in subsequent co-encapsulation studies to promote probiotic growth while avoiding inhibitory effects. As the two polyphenols were tested separately, the observed concentration-dependent dual effects highlight the importance of dosage optimization for each individual polyphenol in co-encapsulation design.

### 3.3. X-Ray Diffraction Analysis

**Figure 2:**
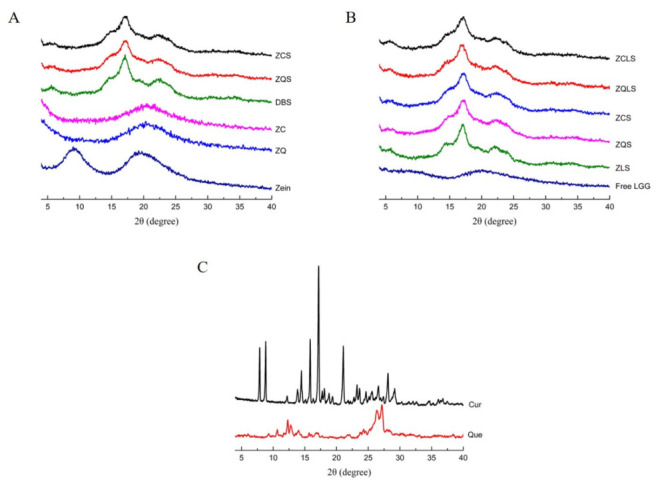
X-ray diffraction pattern of (**A**) single encapsulated particles; (**B**) co-encapsulated particles; and (**C**) free curcumin and quercetin. Cur: Curcumin; Que: Quercetin; Free LGG: Free *Lactobacillus rhamnosus* GG; Zein: Zein; DBS: Debranched starch; ZC: Zein-Cur encapsulated particles; ZQ: Zein-Que encapsulated particles; ZCS: Zein-Cur-DBS encapsulated particles; ZQS: Zein-Que-DBS encapsulated particles; ZLS: Zein-LGG-DBS encapsulated particles; ZCLS: Zein-Cur-LGG-DBS co-encapsulated particles; ZQLS: Zein-Que-LGG-DBS co-encapsulated particles.

The X-ray diffraction patterns of the raw materials and the encapsulated particles are shown in Figure 2. The XRD pattern of free curcumin (Cur) exhibited strong, sharp diffraction peaks at 2θ = 8.8°, 12.2°, 17.2°, 21.1°, and 24.6°, confirming its highly crystalline nature, consistent with previous reports [19,20]. Similarly, free quercetin (Que) displayed distinct sharp diffraction peaks near 2θ = 10.76°, 12.44°, 14.07°, 17.19°, 26.52°, and 27.31°, indicating a well-defined crystalline structure (Figure 2C).

In all encapsulated particles, including zein-only particles (ZC, ZQ), single encapsulated particles (ZCS, ZQS, ZLS), and co-encapsulated particles (ZCLS, ZQLS), the characteristic diffraction peaks of both Cur and Que disappeared entirely (Figure 2A,B). This absence provides direct evidence that the crystalline polyphenols were successfully encapsulated and transformed into an amorphous state within the particle matrix. This transformation is attributed primarily to hydrophobic interactions between the polyphenols and zein during the solvent displacement process. These interactions incorporate the polyphenols into the hydrophobic core formed by zein self-assembly, effectively disrupting their crystal lattice and preventing recrystallization. The transition to an amorphous state is highly beneficial, as amorphous bioactive compounds typically exhibit higher internal energy and specific volume, which can enhance their solubility, dispersibility, and ultimately, their bioavailability [21,22]. The complete disappearance of the characteristic crystalline peaks of curcumin and quercetin in the encapsulated particles, and the presence of only a dominant broad amorphous peak, is in agreement with the XRD patterns reported for their amorphous forms [23,24,25]. This confirms the successful amorphization of both polyphenols within the encapsulation matrix. Furthermore, as quantified in Table 2, the relative crystallinity of the encapsulated particles was significantly lower than that of the debranched starch (DBS) alone. DBS had a crystallinity of 30.84%, whereas values for ZCS, ZQS, ZCLS, and ZQLS dropped to 9.87%, 9.52%, 11.59%, and 12.24%, respectively. This marked reduction confirms that during the electrostatic complex, zein and DBS/chitosan formed a relatively loose and amorphous composite structure through electrostatic interactions. This process disrupts the regular packing of DBS molecules, thereby reducing their long-range structural order. The slightly higher crystallinity of ZCLS and ZQLS compared to ZCS and ZQS might be related to the presence of LGG, which could introduce minor structural variations during co-assembly process. These structural changes, including the transformation to an amorphous state and the reduction in crystallinity, are critical for enhancing the solubility and bioavailability of the polyphenols. Furthermore, the amorphous state of the polyphenols, confirmed by the complete disappearance of their characteristic crystalline peaks, provides a structural basis for the high encapsulation efficiency and yield reported in Section 3.1. This improvement provides a foundation for the enhanced functional performance observed in the encapsulated systems.

### 3.4. Fourier Transform Infrared Spectroscopy Analysis

**Figure 3:**
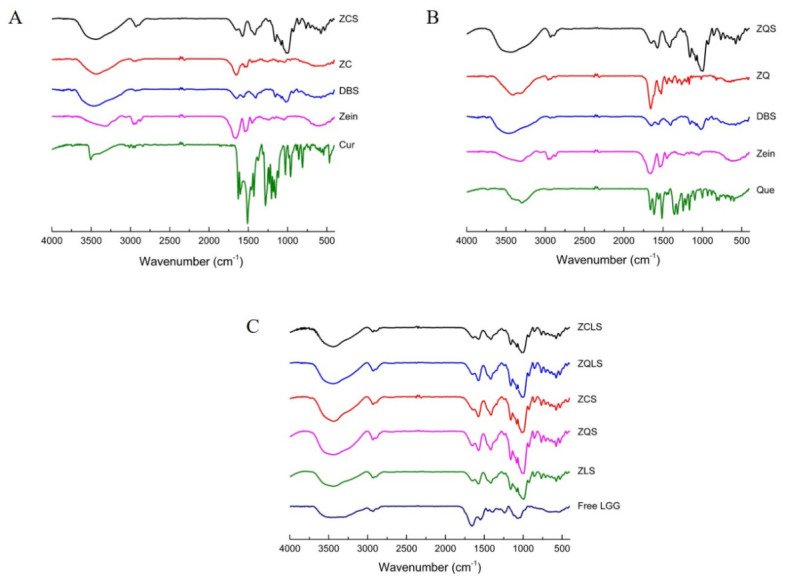
FTIR spectra of (**A**) curcumin and its encapsulated particles; (**B**) quercetin and its encapsulated particles; and (**C**) co-encapsulated particles. Cur: Curcumin; Que: Quercetin; Free LGG: Free *Lactobacillus rhamnosus* GG; Zein: Zein; DBS: Debranched starch; ZC: Zein-Cur particles; ZQ: Zein-Que particles; ZCS: Zein-Cur-DBS particles; ZQS: Zein-Que-DBS particles; ZLS: Zein-LGG-DBS particles; ZCLS: Zein-Cur-LGG-DBS particles; ZQLS: Zein-Que-LGG-DBS particles.

FT-IR spectroscopy was used to investigate molecular interactions among the components in the encapsulated particles. As shown in Figure 3, the spectra of free Cur and Que matched those of their enol forms, as previously reported [26]. Cur exhibited characteristic absorption peaks at 3505 cm^−1^ (O–H stretching), 1629 cm^−1^ (C=O and C=C vibration), 1603 and 1509 cm^−1^ (aromatic ring stretching), 1431 cm^−1^ (C–H bending), 1282 cm^−1^ (aromatic C–O stretching), and 1020 cm^−1^ (C–O–C stretching) (Figure 3A). Que displayed a similar profile in the 1000–1600 cm^−1^ range, with notable peaks at 1664 cm^−1^ (C=O stretching), 1320 cm^−1^ (aromatic C–O stretching), and 1247 cm^−1^ (C–O–C stretching) (Figure 3B). The broader and more intense O–H stretching band around 3300 cm^−1^ for Que compared to Cur reflects its higher number of hydroxyl groups [27]. Zein showed typical protein absorption bands at 3304 cm^−1^ (O–H/N–H stretching), 2935 cm^−1^ (C–H stretching), 1661 cm^−1^ (amide I, C=O stretching), and 1539 cm^−1^ (amide II, N–H bending) (Figure 3A) [14]. The FTIR spectra of the individual raw materials and their corresponding physical mixtures are presented in Supplementary Figure S1 for comparison. When compared with the direct baseline spectra of the encapsulated particles shown in Figure 3, the physical mixture (ZC-1, ZQ-1, ZCS-1, ZQS-1, ZCLS-1, ZQLS-1) exhibited detectable signals originating from free Cur, Que, or LGG. In contrast, no such signals were observed in the spectra of the encapsulated particles. This absence confirms that the outer shell composed of debranched starch and chitosan effectively encapsulated the zein–polyphenol core along with the probiotic, thereby forming an effective physical barrier. In the spectrum of the ZC complex, the characteristic peaks of Cur nearly vanished, indicating effective encapsulation within the zein matrix, primarily mediated by hydrophobic interactions (Figure 3A). In contrast, the ZQ complex still exhibited weak residual Que signals (Figure 3B). This is attributed to the higher polarity of Que, which results from its greater hydroxyl content, enabling interactions not only with hydrophobic zein regions but also with hydrophilic sites, leading to partial surface adsorption on the zein nanoparticles.

The spectra of the composite particles (ZCS, ZQS, ZCLS, ZQLS) showed no detectable signals from free Cur, Que, or LGG, indicating that the outer shell of debranched starch and chitosan successfully encapsulated the zein–polyphenol core and the probiotic, forming an effective physical barrier (Figure 3C). The spectral profiles of these composite particles closely resembled that of DBS, with no new absorption bands emerging, confirming that the encapsulation process relied on physical interactions such as electrostatic complexation, without the formation of covalent bonds. Furthermore, short-range molecular order parameters derived from the FT-IR spectra (Table 2) provided additional structural insight. The double helix degree (DD, measured as the 995/1022 cm^−1^ ratio) was significantly lower in all encapsulated particles than in native DBS. This reduction suggests that electrostatic complexation between DBS and zein/chitosan disrupted the reorganization of DBS linear chains, thereby suppressing the formation of double-helical structures during retrogradation. This result aligns well with the XRD data, which indicated a pronounced loss of long-range crystallinity. Collectively, the XRD and FT-IR analyses confirm that the encapsulation process not only alters the physical state of the polyphenols but also modifies the supramolecular structure of the wall materials. These structural changes are fundamental to achieving high encapsulation efficiency and improved functional performance in the co-encapsulated systems. Specifically, the absence of detectable polyphenol signals in the composite particle spectra, together with the disrupted short-range molecular order indicated by reduced DD values, further supports the effective encapsulation and structural integrity of the co-encapsulated particles as reported in Section 3.1.

### 3.5. Environmental Scanning Electron Microscopy Analysis

**Figure 4:**
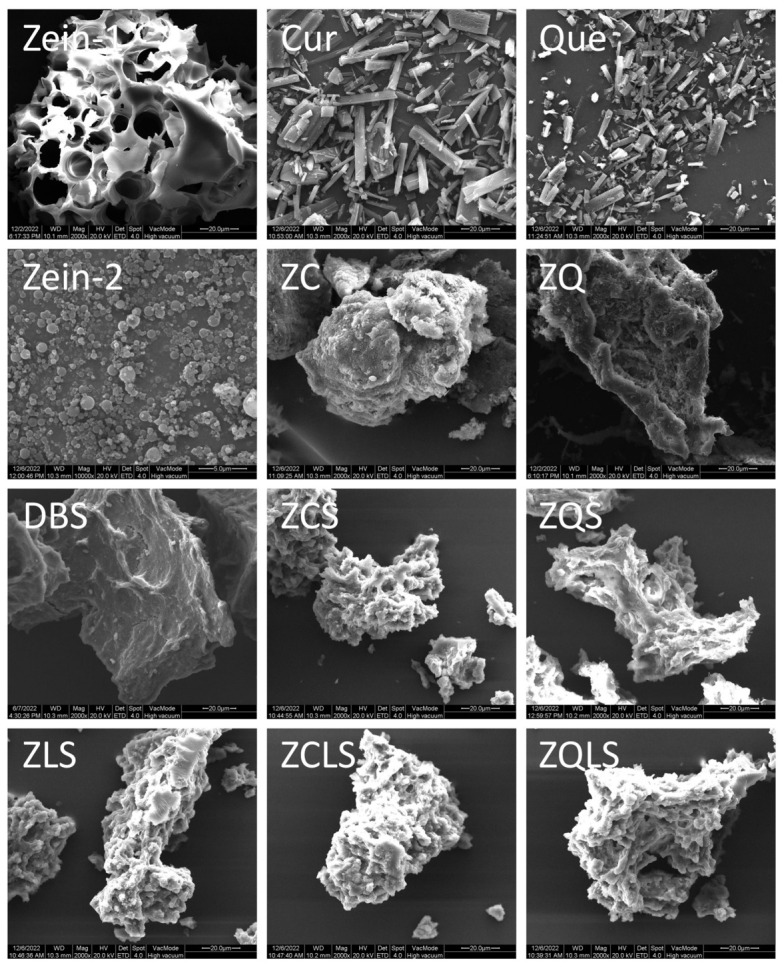
Scanning electron micrographs of encapsulated particles. Cur: Curcumin; Que: Quercetin; Zein-1: Natural zein; Zein-2: Zein particles prepared by solvent displacement method; DBS: Debranched starch; ZC: Zein-Cur particles; ZQ: Zein-Que particles; ZCS: Zein-Cur-DBS particles; ZQS: Zein-Que-DBS particles; ZLS: Zein-LGG-DBS particles; ZCLS: Zein-Cur-LGG-DBS particles; ZQLS: Zein-Que-LGG-DBS particles. Note: All images were captured at ×2000 magnification, except for Zein-1 (×10,000).

The morphological features of the raw materials and encapsulated particles were examined using environmental scanning electron microscopy (ESEM), as shown in Figure 4. Natural zein (Zein-1) exhibited a network-like porous structure, whereas after the solvent displacement treatment, it self-assembled into nanoscale spherical particles (Zein-2), forming a hydrophobic core for polyphenol loading. Free curcumin (Cur) and quercetin (Que) both appeared as irregular, micron-sized crystalline blocks.

Notably, the surface of ZQ particles showed visibly more adsorbed quercetin compared to the amount of curcumin observed on ZC particles. This provides direct visual evidence supporting the FT-IR analysis and the lower encapsulation efficiency recorded for Que (Table 1). It confirms that the more hydrophobic curcumin is effectively incorporated into the zein core, whereas the more polar quercetin tends to adsorb onto the particle surface. Furthermore, curcumin-loaded particles (ZC and ZCS) were generally smaller in size than those loaded with quercetin (ZQ and ZQS). This size difference can be explained by the stronger hydrophobic interaction between the less polar curcumin and zein, promoting the formation of a more compact and dense core during self-assembly.

**Figure 5:**
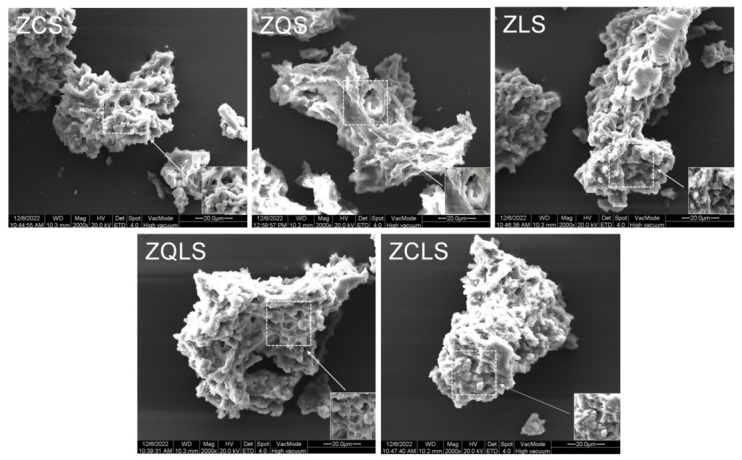
Composite particles (ZCS, ZQS, ZLS, ZCLS, ZQLS) with the magnified insets indicated in Figure 4. ZCS: Zein-Cur-DBS particles; ZQS: Zein-Que-DBS particles; ZLS: Zein-LGG-DBS particles; ZCLS: Zein-Cur-LGG-DBS particles; ZQLS: Zein-Que-LGG-DBS particles. Note: The circled insets for ZCS, ZQS, ZLS, ZCLS, and ZQLS show magnified views of the micron-sized surface aggregates.

In contrast, the composite particles (ZCS, ZQS, ZLS, ZCLS, ZQLS) displayed a morphology that distinctly differed from that of their individual components. The larger particles (exceeding 100 µm) were composed of densely assembled micron-sized aggregates, which exhibited relatively smooth surfaces, as shown in the magnified insets of Figure 5. These images clearly reveal the micron-sized surface aggregates that assemble into the larger particles. This uniform microstructure confirms the successful construction of a composite system through electrostatic complexation among zein, debranched starch, and chitosan, a finding consistent with earlier reports on similar structures formed by zein and chitosan [28]. The observed morphology reflects a clear hierarchical organization, with the outer polysaccharide layer fully enclosing the inner core components. The morphological differences between ZC and ZQ particles, along with the observed surface adsorption of quercetin, help explain the variations in encapsulation behavior between the two polyphenols. These findings emphasize the influence of polyphenol polarity on the assembly and overall performance of the encapsulation system.

### 3.6. Confocal Laser Scanning Microscopy Analysis

Confocal laser scanning microscopy (CLSM) was employed to visualize the spatial distribution of different components within the particles using specific fluorescent dyes, with FITC staining starch green and Rhodamine B staining proteins red. According to established methodology [16], the merged images of red and green signals result in yellow areas, indicating the co-localization of protein and starch.

**Figure 6:**
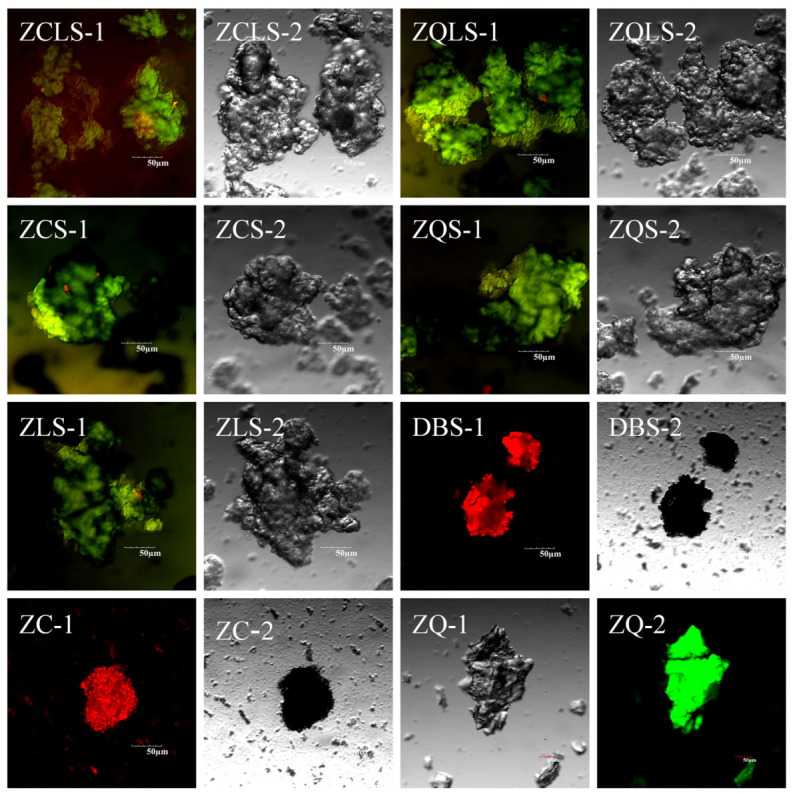
CLSM images of encapsulated particles. For each sample, the left panel 1 displays the merged fluorescence image (FITC, green, starch; Rhodamine B, red, protein), where yellow regions indicate co-localization of starch and protein. The right panel 2 displays the corresponding bright-field image as a morphological reference. DBS: Debranched starch; ZC: Zein-Cur particles; ZQ: Zein-Que particles; ZCS: Zein-Cur-DBS particles; ZQS: Zein-Que-DBS particles; ZLS: Zein-LGG-DBS particles; ZCLS: Zein-Cur-LGG-DBS particles; ZQLS: Zein-Que-LGG-DBS particles.

As shown in Figure 6, DBS alone appeared as green blocky structures. The zein-polyphenol particles (ZC and ZQ) were stained red, confirming their protein-based composition. In striking contrast, all composite particles (ZCS, ZQS, ZLS, ZCLS, and ZQLS) exhibited a dominant and homogeneous yellow color in the merged images. This uniform yellow fluorescence demonstrates that the debranched starch (shown in green) thoroughly coated the zein-based core (shown in red), forming a continuous outer layer. This provides direct visual evidence of the core–shell hierarchical structure, which is essential for the simultaneous encapsulation of hydrophobic polyphenols within the zein core and hydrophilic probiotics in the starch/chitosan matrix. This finding corroborates the successful encapsulation indicated by the XRD and FT-IR results and aligns with observations from other studies on similar systems. The well-defined core–shell structure visualized by CLSM directly explains the high encapsulation efficiency and the enhanced protective effects observed in subsequent stability and simulated gastrointestinal tolerance tests. The well-defined core–shell structure visually confirms the hierarchical design and offers a structural basis for the enhanced protection and controlled release of both polyphenols and probiotics.

### 3.7. Antioxidant Capacity of Curcumin and Quercetin in Encapsulated Particles

The antioxidant properties of curcumin and quercetin are derived from phenolic hydroxyl groups and enol structures present in their molecular frameworks [29]. To evaluate the antioxidant capacity of both free and encapsulated polyphenols, and to compare single- and co-encapsulated systems, DPPH and ABTS radical scavenging assays were conducted.

**Figure 7:**
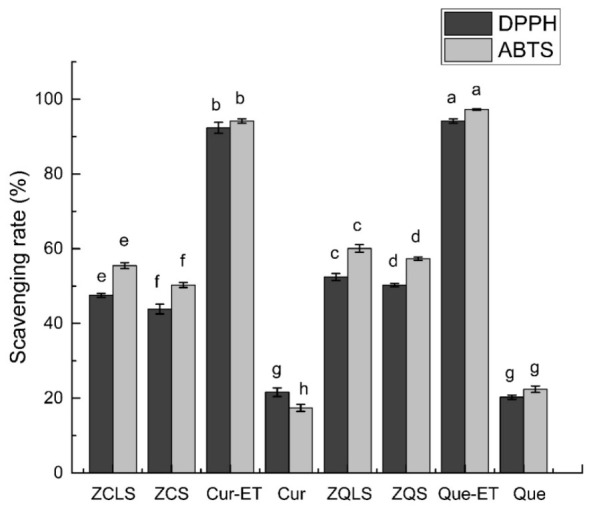
Radical scavenging ability of free compounds and encapsulated particles. ZCLS: Zein-Cur-LGG-DBS particles; ZCS: Zein-Cur-DBS particles; Cur-ET: Curcumin ethanol solution; Cur: Crystalline curcumin; Que-ET: Quercetin ethanol solution; Que: Crystalline quercetin; ZQLS: Zein-Que-LGG-DBS particles; ZQS: Zein-Que-DBS particles. Note: Different lowercase letters indicate significant differences (*p* < 0.05) as determined by one-way ANOVA followed by Duncan’s multiple range test.

As illustrated in Figure 7, ethanolic solutions of curcumin and quercetin displayed radical scavenging rates exceeding 90%. In contrast, their free crystalline forms showed only approximately 20% activity, primarily due to poor solubility and restricted access to radicals. Encapsulation markedly enhanced the antioxidant activity of both compounds, indicating improved dispersibility and solubility within the particle matrix, a result which is consistent with previous study [30]. Regarding the comparison between quercetin and curcumin, Figure 7 shows that in the DPPH assay, the scavenging rates of ZQS (79.3%) and ZQLS (86.1%) were not statistically different from those of ZCS (78.1%) and ZCLS (85.4%) (*p* > 0.05), respectively. However, in the ABTS assay, quercetin-containing particles exhibited significantly higher activity than curcumin-containing ones (*p* < 0.05). This discrepancy may be attributed to the distinct reaction mechanisms and kinetics of the two radicals: the DPPH assay proceeds via a slower hydrogen atom transfer mechanism, whereas the ABTS assay involves faster sequential electron transfer pathways. The faster kinetics and broader mechanistic coverage of the ABTS assay may better capture the full antioxidant potential conferred by quercetin’s multiple hydroxyl groups, while the slower kinetics of the DPPH assay may mask such differences.

Quercetin consistently exhibited stronger antioxidant capacity than curcumin, which can be attributed to its higher number of hydroxyl groups. Notably, the co-encapsulated particles (ZCLS and ZQLS) achieved significantly higher radical scavenging rates than their single-encapsulated counterparts (ZCS and ZQS). This suggests that the presence of LGG may further enhance the polyphenols’ antioxidant activity. Such an interaction underscores the potential for mutualistic effects within co-encapsulation systems. The significantly enhanced antioxidant activity observed in encapsulated systems is closely related to the structural changes revealed by XRD and FT-IR analyses. The transformation of polyphenols from crystalline to amorphous state, along with the well-defined core–shell structure, improves their dispersibility and accessibility to free radicals, thereby enhancing radical scavenging capacity. Specifically, the amorphization of curcumin and quercetin, as confirmed by the complete disappearance of their characteristic crystalline peaks in XRD patterns (Section 3.3), directly contributes to their increased solubility and radical scavenging activity in the encapsulated form.

It should be noted that in the antioxidant assays, the polyphenol content in the encapsulated particles was determined spectrophotometrically as described in Section 2.5, and the assay samples were prepared to contain the same amount of polyphenols as the free polyphenol controls. The observed enhancement in radical scavenging activity therefore reflects improved dispersibility and protection of the polyphenols provided by the encapsulation matrix. A limitation of this study is that the antioxidant activity of the individual wall materials (zein, debranched starch, chitosan) and LGG was not separately evaluated. Furthermore, zein forms non-covalent interactions with the encapsulated polyphenols, and such protein–polyphenol interactions have been shown to partially mask the total antioxidant capacity of the resulting complexes [31]. Consequently, the net antioxidant activity of the co-encapsulated particles likely reflects a balance between the protective benefits offered by encapsulation and the potential masking of polyphenol antioxidant capacity due to interactions with the proteinaceous wall material.

### 3.8. Tolerance in Simulated Gastrointestinal Fluids

**Figure 8:**
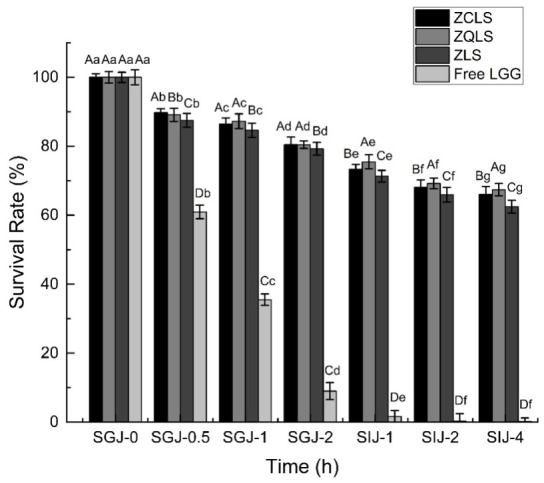
Gastrointestinal tolerance of encapsulated particles and free LGG in vitro. SGJ-0, SGJ-0.5, SGJ-1, SGJ-2: 0, 0.5, 1, 2 h in simulated gastric juice; SIJ-1, SIJ-2, SIJ-4: 1, 2, 4 h in simulated intestinal juice; ZCLS: Zein-Cur-LGG-DBS particles; ZQLS: Zein-Que-LGG-DBS particles; ZLS: Zein-LGG-DBS particles; Free LGG: Free *Lactobacillus rhamnosus* GG. Note: Different uppercase letters within the same time point indicate significant differences among samples (*p* < 0.05). Different lowercase letters within the same sample indicate significant differences over time (*p* < 0.05) as determined by one-way ANOVA followed by Duncan’s multiple range test.

For probiotics to exert their beneficial effects, they must survive transit through the gastrointestinal tract. The tolerance of encapsulated LGG under simulated gastrointestinal conditions was evaluated to assess the protective efficacy of the encapsulation system. As depicted in Figure 8, free LGG viability declined rapidly, with nearly complete inactivation after 2 h in simulated gastric juice (SGJ) and 4 h in simulated intestinal juice (SIJ). In contrast, all encapsulated particles maintained LGG viability above 60% under the same conditions, demonstrating effective protection by the zein-DBS-chitosan matrix. Similar protective effects were reported by Riaz et al. [32] using zein-alginate systems. Importantly, co-encapsulated particles (ZCLS and ZQLS) provided superior protection compared to LGG-only particles (ZLS), with quercetin-based particles (ZQLS) showing the highest survival rates. This suggests that the co-encapsulated polyphenols, particularly quercetin, contribute additional protective effects, possibly through antioxidant mechanisms that mitigate oxidative stress in the gastrointestinal environment. These findings align with Lamprecht et al. [33], who reported that green tea extract co-encapsulated with probiotics reduced gastric acid-induced damage. The superior protective effect of the encapsulation system on LGG viability under simulated gastrointestinal conditions is supported by the structural evidence from multiple characterization techniques. The core–shell hierarchy visualized by CLSM, combined with the amorphous composite structure indicated by XRD, creates an effective barrier against gastric acid and bile salts, while the presence of co-encapsulated polyphenols provides additional protection through their antioxidant properties. It should be noted that a control group consisting of unencapsulated LGG mixed with free curcumin or quercetin was not included in the present gastrointestinal simulation. Future studies incorporating such controls would help to further determine whether direct extracellular interactions between polyphenols and bacteria also contribute to the observed protective effects.

It should be acknowledged that in this simulated gastrointestinal study, the encapsulated particles were exposed directly to gastric juice without undergoing an oral mastication phase. This experimental design was intentional, as the co-encapsulated particles are intended for potential application as a dry powder formulation that may bypass significant mechanical chewing. However, in realistic consumption scenarios where the particles are incorporated into solid or semi-solid food matrices, mastication could partially disrupt the particle structure, leading to premature release of some encapsulated polyphenols and LGG in the oral cavity. This early release might reduce the amount of bioactives reaching the lower gastrointestinal tract and alter the protective profile of the encapsulation system. The presence of saliva, salivary amylase, and mechanical shear could potentially degrade the outer starch/chitosan coating to some extent, thereby affecting the subsequent gastric tolerance. Future studies incorporating a complete simulated oral phase are warranted to quantitatively assess the impact of mastication on the release kinetics of both polyphenols and probiotics from this co-encapsulation system.

### 3.9. Storage Stability

**Figure 9:**
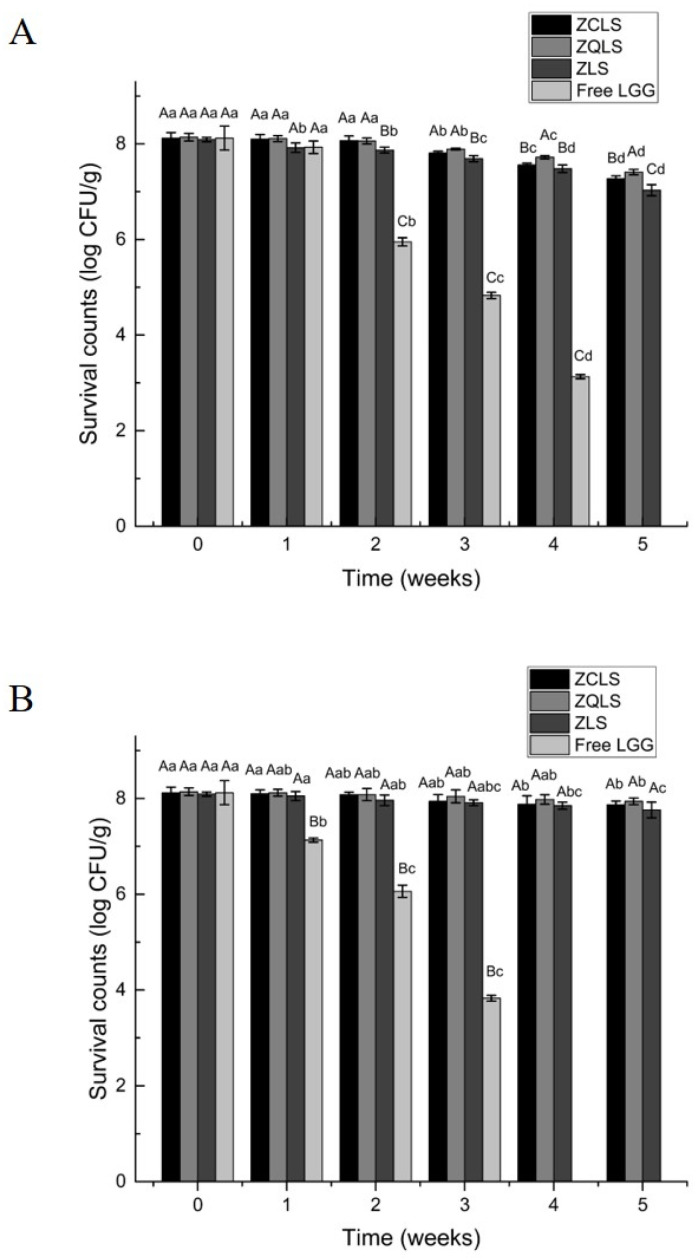
Storage stability of encapsulated particles and free LGG at (**A**) 4 °C; and (**B**) −20 °C. ZCLS: Zein-Cur-LGG-DBS particles; ZQLS: Zein-Que-LGG-DBS particles; ZLS: Zein-LGG-DBS particles; Free LGG: Free *Lactobacillus rhamnosus* GG. Note: Different uppercase letters within the same time point indicate significant differences among samples (*p* < 0.05). Different lowercase letters within the same sample indicate significant differences over time (*p* < 0.05) as determined by one-way ANOVA followed by Duncan’s multiple range test.

The storage stability of encapsulated *Lactobacillus rhamnosus* GG (LGG) was evaluated by monitoring viable cell counts under refrigeration (4 °C) and freezing (–20 °C) conditions, in order to assess the protective effect of the encapsulation system and the potential influence of co-encapsulated curcumin or quercetin on probiotic viability. The results are summarized in Figure 9. With extended storage time, all groups exhibited a general decline in LGG viability. Free LGG was the most susceptible, losing all viability by the end of the storage period. In contrast, encapsulated LGG in ZCLS, ZQLS, and ZLS particles all retained viability above 7.0 log CFU/g. After 5 weeks of storage at 4 °C, the reductions in viable counts were 0.85, 0.73, and 1.06 log CFU/g, respectively, while at −20 °C, the reductions were markedly smaller, at 0.25, 0.20, and 0.33 log CFU/g. These results confirm that the encapsulation system provided substantial protection to LGG, with greater stability observed at −20 °C. Furthermore, the smallest decline in viability occurred in quercetin-containing co-encapsulated particles (ZQLS), followed by curcumin-loaded particles (ZCLS), while LGG-only particles (ZLS) showed the greatest reduction. This indicates that both polyphenols enhanced the storage stability of LGG, with quercetin conferring a significantly stronger protective effect than curcumin. These observations are consistent with the gastrointestinal tolerance results, and together with the previously noted enhancement of polyphenol antioxidant activity by LGG, suggest a mutually beneficial interaction achieved through co-encapsulation. Notably, after 5 weeks of storage, the viability of LGG in all encapsulated groups remained above 6 log CFU/g (exceeding 10^7^ CFU/g), which is well above the generally recommended minimum for probiotic efficacy (10^6^ CFU/g). The enhanced effect of co-encapsulating curcumin and LGG has also been reported by Su et al. [34], who demonstrated improved chemical stability of curcumin and enhanced probiotic survival during long-term storage. The superior protective effect of quercetin on LGG, consistently observed in both simulated gastrointestinal and storage stability tests, confirms its critical role in promoting probiotic survival. This effect likely arises from its potent antioxidant capacity and potential prebiotic properties, which together enhance the synergistic benefits achieved through the co-encapsulation strategy.

## 4. Conclusions

This study successfully established a hierarchical co-encapsulation system by utilizing zein self-assembly to form a hydrophobic core for curcumin or quercetin, followed by complex coacervation with debranched starch and chitosan to construct a hydrophilic outer layer encapsulating LGG. Structural characterization, including X-ray diffraction and confocal microscopy, confirmed the formation of a well-defined core–shell architecture and the transition of polyphenols from a crystalline to an amorphous state. The resulting particles exhibited high encapsulation efficiency and yield for both polyphenols and probiotics, with values exceeding 95% and 80% for polyphenols, and approximately 95% and 75% for LGG, respectively. Encapsulation significantly enhanced the antioxidant capacity of both curcumin and quercetin, and this effect was further amplified when co-encapsulated with LGG. Notably, the hierarchical structure provided substantial protection to LGG, significantly improving its viability under simulated gastrointestinal conditions and during long-term storage at 4 °C and −20 °C, with quercetin demonstrating a stronger protective effect than curcumin. These findings together validate the efficacy of this co-encapsulation strategy in achieving mutual stabilization and functional enhancement between probiotics and polyphenols, offering a highly effective and adaptable approach for applications in functional foods and nutraceuticals.

## Figures and Tables

**Table 1:** Encapsulation efficiency and yield of encapsulated particles.

Samples	EE (Cur/Que, %)	EY (Cur/Que, %)	EE (LGG, %)	EY (LGG, %)
ZC	84.28 ± 0.25 ^d^	71.26 ± 0.61 ^c^	–	–
ZQ	72.49 ± 0.21 ^e^	65.12 ± 0.21 ^d^	–	–
ZCS	96.21 ± 0.33 ^ab^	80.27 ± 0.61 ^b^	–	–
ZQS	95.19 ± 0.45 ^c^	80.45 ± 0.24 ^b^	–	–
ZLS	–	–	94.18 ± 0.10 ^b^	76.36 ± 0.30 ^c^
ZCLS	96.56 ± 0.28 ^a^	81.73 ± 0.66 ^a^	94.87 ± 0.28 ^a^	78.39 ± 0.33 ^b^
ZQLS	95.64 ± 0.48 ^bc^	80.79 ± 0.42 ^b^	95.09 ± 0.50 ^a^	79.72 ± 0.69 ^a^

Values are expressed as mean ± standard deviation. Different lowercase superscript letters within the same column indicate significant differences (*p* < 0.05) as determined by one-way ANOVA followed by Duncan’s multiple range test. EE: Encapsulation Efficiency; EY: Encapsulation Yield. The symbol “–” indicates that the parameter is not applicable for the corresponding sample. ZC: Zein-Cur particles; ZQ: Zein-Que particles; ZCS: Zein-Cur-DBS particles; ZQS: Zein-Que-DBS particles; ZLS: Zein-LGG-DBS particles; ZCLS: Zein-Cur-LGG-DBS particles; ZQLS: Zein-Que-LGG-DBS particles.

**Table 2:** Multi-scale structural parameters of raw materials and encapsulated particles.

Sample	DO (1047/1022 cm^−1^)	DD (995/1022 cm^−1^)	Relative Crystallinity (XRD, C1, %)
ZCLS	1.0266 ± 0.0003 ^d^	1.0012 ± 0.0002 ^c^	11.59 ± 0.23 ^c^
ZQLS	1.0428 ± 0.0002 ^b^	1.0023 ± 0.0001 ^b^	12.24 ± 0.46 ^c^
ZCS	1.0379 ± 0.0007 ^c^	0.9955 ± 0.0002 ^d^	9.87 ± 0.04 ^d^
ZQS	1.0557 ± 0.0003 ^a^	0.9880 ± 0.0007 ^e^	9.52 ± 0.48 ^d^
ZLS	1.0256 ± 0.0003 ^e^	0.9868 ± 0.0002 ^f^	13.76 ± 0.29 ^b^
DBS	1.0258 ± 0.0008 ^de^	1.0177 ± 0.0005 ^a^	30.84 ± 0.18 ^a^

Values are expressed as mean ± standard deviation. Different lowercase superscript letters within the same column denote significant differences (*p* < 0.05) as determined by one-way ANOVA followed by Duncan’s multiple range test. DBS: Debranched starch; ZLS: Zein-LGG-DBS encapsulated particles; ZCS: Zein-Cur-DBS encapsulated particles; ZQS: Zein-Que-DBS encapsulated particles; ZCLS: Zein-Cur-LGG-DBS co-encapsulated particles; ZQLS: Zein-Que-LGG-DBS co-encapsulated particles.

## Data Availability

The original contributions presented in the study are included in the article/Supplementary Materials, further inquiries can be directed to the corresponding author.

## Supplementary Materials

The following supporting information can be downloaded at: https://www.mdpi.com/article/10.3390/foods15111910/s1, Figure S1: Control FTIR spectra of physical mixtures of the individual raw materials. ZC-1: physical mixture of zein and curcumin; ZQ-1: physical mixture of zein and quercetin; ZCS-1: physical mixture of zein, curcumin, and debranched starch; ZQS-1: physical mixture of zein, quercetin, and debranched starch; ZLS-1: physical mixture of zein, LGG, and debranched starch; ZCLS-1: physical mixture of zein, curcumin, LGG, and debranched starch; ZQLS-1: physical mixture of zein, quercetin, LGG, and debranched starch.

## References

[1] Geng T., He F., Su S., Sun K., Zhao L., Zhao Y., Bao N., Pan L., Sun H. (2021). Probiotics Lactobacillus Rhamnosus GG ATCC53103 and Lactobacillus Plantarum JL01 Induce Cytokine Alterations by the Production of TCDA, DHA, and Succinic and Palmitic Acids, and Enhance Immunity of Weaned Piglets. Res. Vet. Sci..

[2] Chang R., Xu K., Zhang R., Jin Z., Aiguo M. (2024). A Combined Recrystallization and Acetylation Strategy for Resistant Starch with Enhanced Thermal Stability and Excellent Short-Chain Fatty Acid Production. Food Chem..

[3] Liu G., Gu Z., Hong Y., Cheng L., Li C. (2017). Structure, Functionality and Applications of Debranched Starch: A Review. Trends Food Sci. Technol..

[4] Sun R., Lv Z., Wang Y., Li M., Qi J., Wang K., Yang H., Yue T., Yuan Y. (2024). Different Polysaccharide-Enhanced Probiotic and Polyphenol Dual-Functional Factor Co-Encapsulated Microcapsules Demonstrate Acute Colitis Alleviation Efficacy and Food Fortification. Carbohydr. Polym..

[5] Tang Y., Liu W., Zhang J., Juan B., Zhu Y., Zhu L., Zhao Y., Daglia M., Xiao X., He Y. (2025). Advances in Intestinal-Targeted Release of Phenolic Compounds. Nutrients.

[6] Eratte D., McKnight S., Gengenbach T.R., Dowling K., Barrow C.J., Adhikari B.P. (2015). Co-Encapsulation and Characterisation of Omega-3 Fatty Acids and Probiotic Bacteria in Whey Protein Isolate–Gum Arabic Complex Coacervates. J. Funct. Foods.

[7] Eratte D., Dowling K., Barrow C.J., Adhikari B.P. (2017). In-Vitro Digestion of Probiotic Bacteria and Omega-3 Oil Co-Microencapsulated in Whey Protein Isolate-Gum Arabic Complex Coacervates. Food Chem..

[8] Eratte D., Wang B., Dowling K., Barrow C.J., Adhikari B. (2016). Survival and Fermentation Activity of Probiotic Bacteria and Oxidative Stability of Omega-3 Oil in Co-Microcapsules during Storage. J. Funct. Foods.

[9] Wang Y., Padua G.W. (2012). Nanoscale Characterization of Zein Self-Assembly. Langmuir.

[10] Zhang Y., Cui L., Che X., Zhang H., Shi N., Li C., Chen Y., Kong W. (2015). Zein-Based Films and Their Usage for Controlled Delivery: Origin, Classes and Current Landscape. J. Control. Release.

[11] Hu K., Huang X., Gao Y., Huang X., Xiao H., McClements D.J. (2015). Core–Shell Biopolymer Nanoparticle Delivery Systems: Synthesis and Characterization of Curcumin Fortified Zein–Pectin Nanoparticles. Food Chem..

[12] Zhang X., Zheng F., Dai X., Liu J., Xiu L., Liu H., Cai D. (2025). Zein/Ferulic Acid/Sodium Alginate Ternary Nanoparticles: Characterization and Application in Stable Pickering Emulsions. Food Chem..

[13] Xu J., Ma Z., Ren N., Li X., Liu L., Hu X. (2019). Understanding the Multi-Scale Structural Changes in Starch and Its Physicochemical Properties during the Processing of Chickpea, Navy Bean, and Yellow Field Pea Seeds. Food Chem..

[14] Meng R., Wu Z., Xie Q.-T., Cheng J.-S., Zhang B. (2021). Preparation and Characterization of Zein/Carboxymethyl Dextrin Nanoparticles to Encapsulate Curcumin: Physicochemical Stability, Antioxidant Activity and Controlled Release Properties. Food Chem..

[15] Zou Y., Qian Y., Rong X., Cao K., McClements D.J., Hu K. (2021). Encapsulation of Quercetin in Biopolymer-Coated Zein Nanoparticles: Formation, Stability, Antioxidant Capacity, and Bioaccessibility. Food Hydrocoll..

[16] Jia F., Ma Z., Hu X. (2020). Controlling Dough Rheology and Structural Characteristics of Chickpea-Wheat Composite Flour-Based Noodles with Different Levels of Artemisia Sphaerocephala Krasch. Gum Addition. Int. J. Biol. Macromol..

[17] Mukherjee P., Baruah K.N., Uppaluri R.V.S. (2024). Encapsulation and Characterization of Commercial Green Tea Extracts Using Green Methods: A Comparative Study of Inclusion Complexation and Ion Gelation. Food Meas..

[18] Liang J., Yan H., Wang X., Zhou Y., Gao X., Puligundla P., Wan X. (2017). Encapsulation of Epigallocatechin Gallate in Zein/Chitosan Nanoparticles for Controlled Applications in Food Systems. Food Chem..

[19] Li J., Shin G.H., Lee I.W., Chen X., Park H.J. (2016). Soluble Starch Formulated Nanocomposite Increases Water Solubility and Stability of Curcumin. Food Hydrocoll..

[20] Touzout Z., Abdellaoui N., Hadj-Hamou A.S. (2024). Conception of pH-Sensitive Calcium Alginate/Poly Vinyl Alcohol Hydrogel Beads for Controlled Oral Curcumin Delivery Systems. Antibacterial and Antioxidant Properties. Int. J. Biol. Macromol..

[21] Jog R., Burgess D.J. (2017). Pharmaceutical Amorphous Nanoparticles. J. Pharm. Sci..

[22] Ludwig D.B., De Camargo L.E.A., Tominaga T.T., Mainardes R.M. (2025). Chitosan-Coated PLGA Nanospheres for Improved Amphotericin B Delivery: Characterization, Mucoadhesion, and Cytotoxicity Assessment. BioNanoScience.

[23] Dian L., Yu E., Chen X., Wen X., Zhang Z., Qin L., Wang Q., Li G., Wu C. (2014). Enhancing Oral Bioavailability of Quercetin Using Novel Soluplus Polymeric Micelles. Nanoscale Res. Lett..

[24] Li B., Konecke S., Wegiel L.A., Taylor L.S., Edgar K.J. (2013). Both Solubility and Chemical Stability of Curcumin Are Enhanced by Solid Dispersion in Cellulose Derivative Matrices. Carbohydr. Polym..

[25] Shi X., Fan N., Zhang G., Sun J., He Z., Li J. (2020). Quercetin Amorphous Solid Dispersions Prepared by Hot Melt Extrusion with Enhanced Solubility and Intestinal Absorption. Pharm. Dev. Technol..

[26] Lupu A., Bercea M., Avadanei M., Gradinaru L.M., Nita L.E., Gradinaru V.R. (2025). Temperature Sensitive Pluronic F127-Based Gels Incorporating Natural Therapeutic Agents. Macromol. Mater. Eng..

[27] Ma L., Su C.-R., Li S.-Y., He S., Nag A., Yuan Y. (2023). Co-Delivery of Curcumin and Quercetin in the Bilayer Structure Based on Complex Coacervation. Food Hydrocoll..

[28] Cao Y., Yang Y., Liang Z., Guo W., Lv X., Ni L., Chen Y. (2024). Synthesis of Ganoderic Acids Loaded Zein-Chitosan Nanoparticles and Evaluation of Their Hepatoprotective Effect on Mice Given Excessive Alcohol. Foods.

[29] O’Toole M.G., Soucy P.A., Chauhan R., Raju M.V.R., Patel D.N., Nunn B.M., Keynton M.A., Ehringer W.D., Nantz M.H., Keynton R.S. (2016). Release-Modulated Antioxidant Activity of a Composite Curcumin-Chitosan Polymer. Biomacromolecules.

[30] Qin Y., Wang J., Qiu C., Hu Y., Xu X., Jin Z. (2019). Effects of Degree of Polymerization on Size, Crystal Structure, and Digestibility of Debranched Starch Nanoparticles and Their Enhanced Antioxidant and Antibacterial Activities of Curcumin. ACS Sustain. Chem. Eng..

[31] Stojadinovic M., Radosavljevic J., Ognjenovic J., Vesic J., Prodic I., Stanic-Vucinic D., Cirkovic Velickovic T. (2013). Binding Affinity between Dietary Polyphenols and β-Lactoglobulin Negatively Correlates with the Protein Susceptibility to Digestion and Total Antioxidant Activity of Complexes Formed. Food Chem..

[32] Riaz T., Iqbal M.W., Saeed M., Yasmin I., Hassanin H.A.M., Mahmood S., Rehman A. (2019). In Vitro Survival of *Bifidobacterium bifidum* Microencapsulated in Zein-Coated Alginate Hydrogel Microbeads. J. Microencapsul..

[33] Lamprecht A., Schäfer U.F., Lehr C.-M. (2000). Characterization of Microcapsules by Confocal Laser Scanning Microscopy: Structure, Capsule Wall Composition and Encapsulation Rate. Eur. J. Pharm. Biopharm..

[34] Su J., Cai Y., Zhi Z., Guo Q., Mao L., Gao Y., Yuan F., Van Der Meeren P. (2021). Assembly of Propylene Glycol Alginate/β-Lactoglobulin Composite Hydrogels Induced by Ethanol for Co-Delivery of Probiotics and Curcumin. Carbohydr. Polym..

