# Supplementary material for: Preparation and Stability Study of Co-Encapsulated Particles of Curcumin or Quercetin with Lactobacillus rhamnosus GG
Source: Foods. 2026 May 28;15(11):1910. doi: 10.3390/foods15111910 (PMC13257281; doi:10.3390/foods15111910)
Supplement: Supplementary file 1 [file foods-15-01910-s001.zip › foods-4278506-supplementary.pdf]

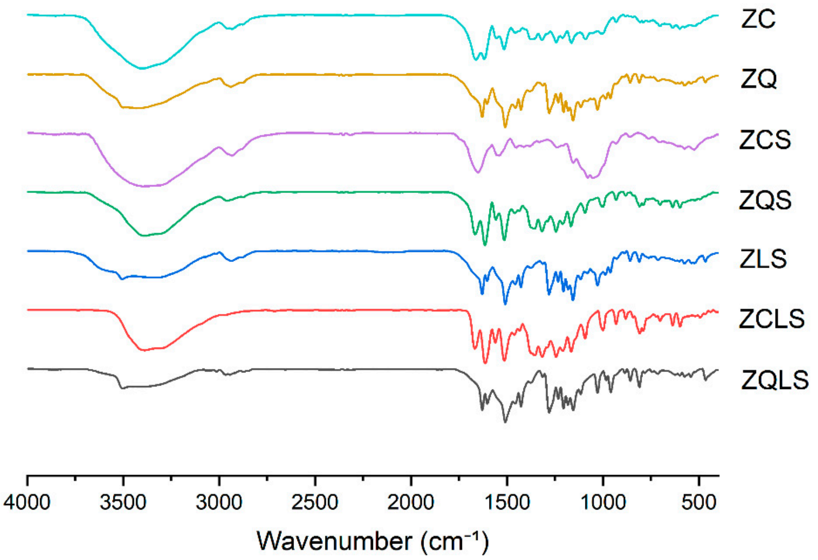

Figure S1 Control FTIR spectra of physical mixtures of the individual raw materials. ZC-1: physical mixture of zein and curcumin; ZQ-1: physical mixture of zein and quercetin; ZCS-1: physical mixture of zein, curcumin, and debranched starch; ZQS-1: physical mixture of zein, quercetin, and debranched starch; ZLS-1: physical mixture of zein, LGG, and debranched starch; ZCLS-1: physical mixture of zein, curcumin, LGG, and debranched starch; ZQLS-1: physical mixture of zein, quercetin, LGG, and debranched starch.
